# TAT-PBX1 Reverses Hyperglycemia Through β-Cell Regeneration and Functional Restoration in an STZ-Induced Diabetic Model

**DOI:** 10.3390/ph19010085

**Published:** 2026-01-01

**Authors:** Xiangyuan Meng, Zhenhu Zhao, Xin Zhang, Ruihan Guo, Shuran Yang, Shuhua Mao, Ziyu Zong, Jinyu Liu

**Affiliations:** School of Public Health, Jilin University, Changchun 130021, China

**Keywords:** diabetes mellitus, STZ-induced diabetes, pancreatic β cells, PBX1, glucokinase

## Abstract

**Objective:** β-cell dysfunction and loss are major pathological determinants of impaired islet function and hyperglycemia in diabetes. Given the inability of current therapies to restore β-cell viability or glucose-responsive insulin secretion, this study aimed to investigate whether a cell-permeable PBX1 fusion protein (TAT-PBX1) could rescue streptozotocin (STZ)-induced β-cell injury and restore β-cell functional integrity. **Methods:** A TAT-PBX1 recombinant fusion protein was produced using a prokaryotic expression system. Its protective effects were assessed in STZ-treated MIN6 β cells and in a mouse model of STZ-induced diabetes, with the glucokinase (GK) activator dorzagliatin included as a positive control. We evaluated β-cell apoptosis, DNA damage, ATP and NAD^+^/NADH levels, insulin signaling (IRS1/PI3K/Akt), and the expression of PDX1 and GK. Glucose-stimulated insulin secretion (GSIS), glucose tolerance, islet morphology, and β-cell proliferation were also examined in vivo. **Results:** TAT-PBX1 was detectable and significantly enriched in pancreatic tissue and mitigated STZ-induced cytotoxicity by reducing DNA damage, PARP1-associated energy depletion, and β-cell apoptosis. It restored intracellular ATP and NAD^+^/NADH ratios and reactivated IRS1/PI3K/Akt signaling. TAT-PBX1 further enhanced PDX1 protein levels and upregulated GK, resulting in improved glucose uptake and GSIS. In addition, it increased Ki67^+^ β-cell proliferation. In diabetic mice, TAT-PBX1 improved glucose tolerance, preserved islet morphology and number, and improved insulin signaling responsiveness. **Conclusions:** TAT-PBX1 restores β-cell function through coordinated protection of cellular metabolism and insulin signaling, leading to improved β-cell survival, glucose responsiveness, and regenerative capacity. These findings support TAT-PBX1 as a promising molecular strategy for β-cell-protective and β-cell-restorative diabetes therapy.

## 1. Introduction

Diabetes has emerged as one of the foremost global public health challenges. According to the International Diabetes Federation (2021), approximately 537 million adults worldwide have diabetes [1]. Although type 1 diabetes mellitus (T1DM) and type 2 diabetes mellitus (T2DM) differ markedly in etiology, both share a convergent pathological endpoint: impairment of pancreatic β-cell function [2]. Loss of β-cell mass together with reduced insulin secretory capacity leads to hyperglycemia, and conversely, hyperglycemia and metabolic stress further damage β cells and worsen systemic metabolic dysfunction, creating a self-reinforcing cycle [3]. Current clinical management primarily targets glycemic control and peripheral insulin sensitivity, but these approaches rarely restore β-cell viability or intrinsic metabolic competence [4]. Therefore, therapeutic strategies that preserve or restore β-cell survival and energy metabolism—thereby supporting both repair mechanisms and energy supply—have potential relevance for both T1DM and T2DM and represent an important public health objective.

β-cell survival is a prerequisite for secretory competence, and maintenance of energy-metabolic homeostasis is critical to support insulin synthesis and release [5]. β cells import and metabolize glucose to generate ATP, which fuels insulin biosynthesis and stimulus-secretion coupling [6]. Glucokinase (GK) serves as the rate-limiting enzyme for β-cell glucose metabolism, phosphorylating glucose to glucose-6-phosphate and initiating downstream glycolysis to promote ATP production [7,8]. Normal transduction of insulin signaling facilitates nuclear translocation and stabilization of the transcription factor PDX1, supporting the expression of genes required for glucose sensing and insulin secretion, including GK, and preserving β-cell glucose sensing and energy supply [9,10]. When energy supply is insufficient or when DNA damage provokes energetic collapse, downstream insulin signaling can be impaired, leading to reduced GK expression, diminished ATP generation, and consequent β-cell dysfunction and apoptosis [11,12]. Streptozotocin (STZ), an N-alkylating agent derived from Streptomyces, is widely used as a chemical model of β-cell injury. STZ enters β cells via Glucose Transporter 2 (GLUT2), induces DNA alkylation and overactivates poly(ADP-ribose) polymerase (PARP), thereby depleting NAD^+^ and ATP and provoking an energetic crisis that further suppresses GK expression and insulin secretion [13,14,15]. The resulting decline in insulin output deepens energy deficiency, completing a vicious cycle that culminates in β-cell death and loss of function. Thus, protecting insulin signaling, restoring GK function, and maintaining β-cell energetic homeostasis are key molecular strategies for mitigating diabetes progression.

Pre-B-cell leukemia homeobox 1 (PBX1) is a homeodomain transcription factor that has been implicated in metabolic balance, energy homeostasis, and DNA damage responses [16]. Epidemiological and genome-wide association studies have linked PBX1 polymorphisms to T2DM, insulin sensitivity, and obesity-related metabolic phenotypes, implicating PBX1 in systemic glucose–lipid homeostasis [17,18,19]. PBX1 is reported to be enriched in mature pancreatic β cells and modulates the Akt/mTOR axis and redox balance, thereby attenuating glucotoxicity-induced mitochondrial dysfunction and β-cell apoptosis [18,19,20]. PBX1 downregulation or loss of function reduces mitochondrial activity, increases oxidative stress, and blunts glucose-stimulated insulin secretion (GSIS), leading to secretory failure and energetic imbalance—features that mirror β-cell decline in T2DM [21,22]. Our prior work indicates that PBX1 and its downstream effector NANOG activate the AKT/Glycogen synthase kinase-3 beta (GSK3β) pathway to sustain energy supply during proliferation and to promote cell growth [23]. Moreover, PBX1 mitigates ROS-mediated DNA damage and energetic depletion, helping to preserve intracellular ATP and metabolic homeostasis and to delay apoptosis and functional decline [24]. Collectively, these studies support the concept that targeting PBX1 to restore β-cell energy metabolism and insulin-signaling competence may represent a novel therapeutic approach for diabetes.

In this work, we evaluate the therapeutic potential of PBX1 in STZ-induced MIN6 cell and mouse models of diabetes, focusing on whether PBX1 can reduce STZ-induced β-cell apoptosis and restore the cellular energy supply to recover β-cell function. To avoid risks associated with viral vectors or genomic integration inherent to gene-editing approaches—risks that are especially pertinent in metabolically vulnerable β cells—we employed cell-penetrating peptide technology (TAT domain) to deliver PBX1 as an exogenous protein. By using a prokaryotic expression system, we generated a TAT-PBX1 fusion protein that preserves PBX1 transcriptional activity while facilitating cellular uptake. To benchmark functional effects on the GK axis in this STZ injury context, we included Dorzagliatin, a glucokinase activator approved for the treatment of T2DM in mainland China, as a positive control [25]. By systematically assessing TAT-PBX1 in cellular and animal models, we aim to provide mechanistic insight and preclinical evidence to inform the development of β-cell-targeted, function-restorative therapies for diabetes.

## 2. Results

### 2.1. Purification of TAT-PBX1

**Figure 1:**
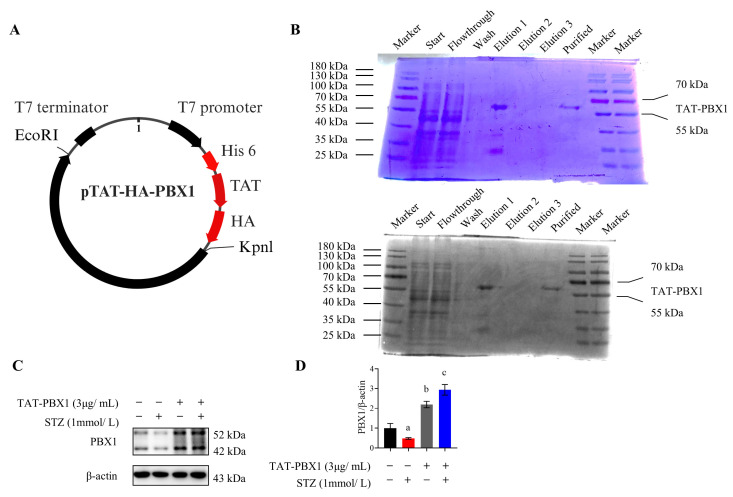
Purification and Cellular Uptake of TAT-PBX1. (**A**) Schematic diagram of the TAT-PBX1 plasmid: the PBX1 DNA sequence was inserted into the pTAT-HA plasmid vector at the C-terminus of the TAT peptide and contains an N-terminal His6 tag for purification and an HA tag for detection. (**B**) Detection of the affinity-purified TAT-PBX1 fusion protein by SDS-PAGE and Coomassie brilliant blue staining. (**C**) Representative Western blot bands of PBX1 levels in MIN6 cell lysates. (**D**) Quantitative analysis of PBX1 levels (*n* = 3). Statistical analyses were performed using Levene’s test for homogeneity of variance. Data meeting homogeneity of variance assumptions were analyzed using one-way ANOVA with the Holm–Šídák multiple comparisons test, while nonhomoscedastic data were evaluated using the Kruskal–Wallis test with Dunn’s multiple comparisons test. a: STZ vs. Control, *p* < 0.05; b: TAT-PBX1 vs. STZ, *p* < 0.05; c: TAT-PBX1 + STZ vs. STZ, *p* < 0.05. Created in BioRender. Meng, X. (2025) https://BioRender.com/jnvv4i5.

A prokaryotic expression construct encoding the TAT-PBX1 fusion protein was successfully generated by cloning the full-length PBX1 coding sequence downstream of the TAT peptide in the pTAT-HA vector. A His6 tag was introduced at the N-terminus for affinity purification, and an HA tag was retained for detection of the fusion protein (Figure 1A). SDS–PAGE analysis revealed a predominant band at ~55 kDa, consistent with the theoretical molecular weight of the fusion protein and indicating robust expression of the target product in *Escherichia coli* (Figure 1B). Following purification, cell-uptake experiments analyzed by Western blot showed that cellular PBX1 signals were significantly increased in MIN6 cells treated with TAT-PBX1 compared with control and STZ-treated groups, consistent with increased cellular PBX1 levels following TAT-PBX1 treatment (Figure 1C,D).

### 2.2. TAT-PBX1 Reduces STZ-Induced DNA Damage and Apoptosis in MIN6 Cells and Partially Restores Energy Supply

**Figure 2:**
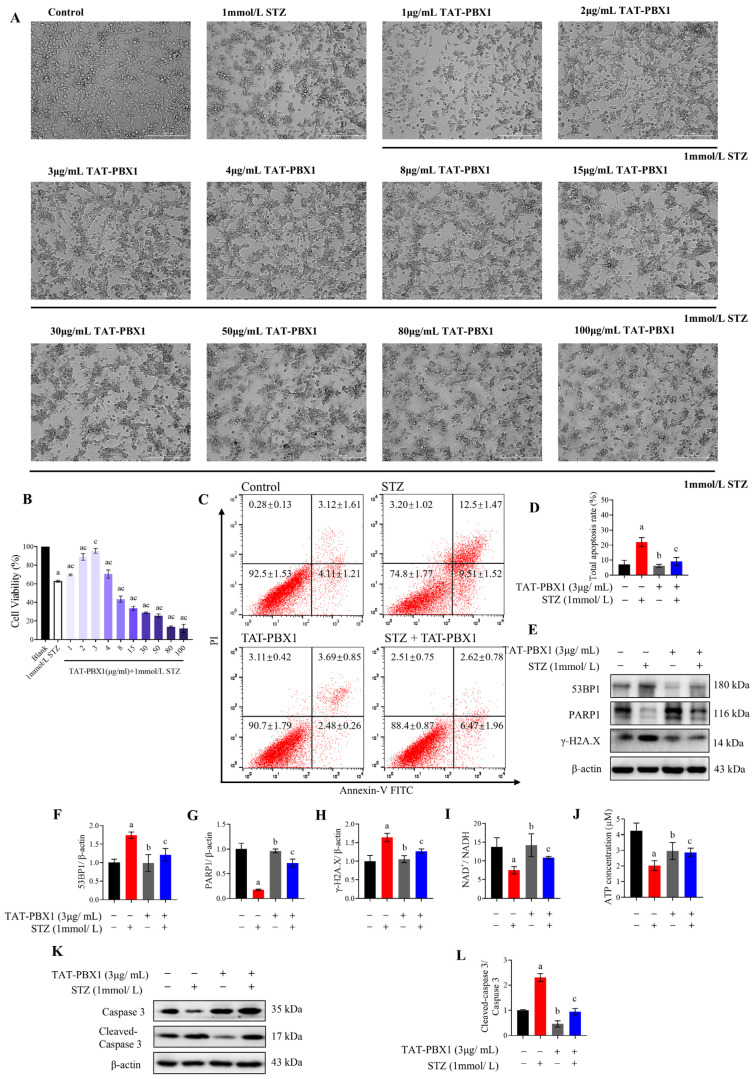
TAT-PBX1 attenuates STZ-induced DNA damage and apoptosis in MIN6 cells and improves cellular energy status. (**A**) Morphology of MIN6 cells treated with STZ alone or co-treated with STZ and TAT-PBX1 under a microscope, scale bar = 200 μm. (**B**) Cell viability of MIN6 cells co-treated with STZ and TAT-PBX1 at concentrations of 1, 2, 3, 4, 8, 15, 30, 50, 80, 100 μg/mL, measured by CCK-8 assay (*n* = 3). (**C**) Apoptosis analysis of MIN6 cells treated with STZ and/or TAT-PBX1 using Annexin V/PI double-staining flow cytometry (*n* = 3). (**D**) Quantification of early (Annexin V^+^/PI^−^) and late (Annexin V^+^/PI^+^) apoptotic cells (*n* = 3). (**E**) Representative Western blot bands of Caspase 3 and cleaved Caspase 3 protein expression. (**F**) Ratio of cleaved Caspase 3 to Caspase 3 (*n* = 3). (**G**) Representative Western blot bands of 53BP1, PARP1, and γ-H2A.X protein expression. (**H**) Quantitative analysis of 53BP1 protein expression (*n* = 3). (**I**) Quantitative analysis of PARP1 protein expression (*n* = 3). (**J**) Quantitative analysis of γ-H2A.X protein expression (*n* = 3). (**K**) NAD^+^/NADH ratio in MIN6 cells (*n* = 3). (**L**) ATP concentration in MIN6 cells (*n* = 3). Statistical analyses were performed using Levene’s test for homogeneity of variance. Data meeting homogeneity of variance assumptions were analyzed using one-way ANOVA with the Holm–Šídák multiple comparisons test, while nonhomoscedastic data were evaluated using the Kruskal–Wallis test with Dunn’s multiple comparisons test. a: STZ vs. Control, *p* < 0.05; b: TAT-PBX1 vs. STZ, *p* < 0.05; c: TAT-PBX1 + STZ vs. STZ, *p* < 0.05.

To validate the survival-protective effect, Annexin V/PI flow cytometry and Western blot analyses were performed. STZ significantly increased the proportions of early and late apoptotic cells relative to control (Figure 2C,D). Compared with the STZ group, co-treatment with TAT-PBX1 significantly reduced total apoptosis, whereas TAT-PBX1 alone did not alter apoptosis relative to control (Figure 2C,D). Western blot analysis showed that STZ increased the cleaved caspase-3/caspase-3 ratio, and co-treatment with TAT-PBX1 reduced this ratio toward control levels (Figure 2E,F). These data indicate that TAT-PBX1 exerts an anti-apoptotic effect in the STZ-injury model without altering baseline apoptosis in healthy cells.

To assess effects on DNA damage and energy metabolism, we measured DNA damage markers and cellular energy indices. STZ (1 mmol/L) upregulated DNA damage markers 53BP1 and γ-H2A.X and was associated with altered PARP1 protein abundance relative to control (Figure 2G–J), consistent with STZ-induced DNA damage and accompanying disturbances in DNA damage response and cellular energetics. Compared with STZ alone, co-treatment with TAT-PBX1 significantly reduced 53BP1 and γ-H2A.X levels and normalized PARP1 expression; TAT-PBX1 alone did not significantly alter these damage markers compared to the control group, suggesting no detectable increase in DNA damage-marker levels under basal conditions (Figure 2G–J). Functionally, STZ markedly decreased the cellular NAD^+^/NADH ratio and ATP content (Figure 2K,L); in STZ-injured cells, TAT-PBX1 significantly increased NAD^+^/NADH and ATP levels, partially or fully restoring them toward control values. Together, these results indicate that TAT-PBX1 attenuates STZ-induced accumulation of DNA damage markers, normalizes PARP1 expression and mitigates NAD^+^/ATP depletion, thereby helping to preserve β-cell energy homeostasis and reduce apoptosis risk associated with energetic collapse.

### 2.3. TAT-PBX1 Restores Insulin Signaling in STZ-Injured MIN6 Cells and Improves Insulin Secretory Function

**Figure 3:**
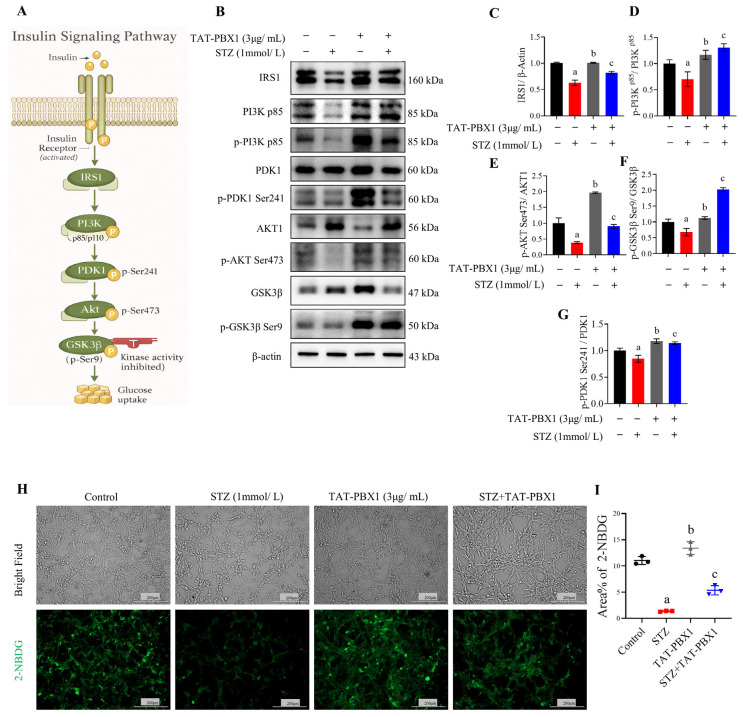
TAT-PBX1 restores insulin signaling in MIN6 cells under STZ treatment. (**A**) Schematic of the canonical IRS1/PI3K/PDK1/Akt/GSK3β signaling axis linked to β-cell metabolic regulation and glucose uptake. (**B**) Representative Western blot bands of IRS1, PI3K p85, p-PI3K p85, p-Akt S473, Akt, p-GSK3β S9, GSK3β, PDK1, and p-PDK1 S241 protein expression after treatments with STZ and/or TAT-PBX1 (*n* = 3). (**C**) Quantitative analysis of IRS1 protein expression (*n* = 3). (**D**) Ratio of p-PI3K p85 to PI3K p85. (**E**) Ratio of p-Akt S473 to Akt. (**F**) Ratio of p-GSK3β S9 to GSK3β. (**G**) Ratio of p-PDK1 S241 to PDK1. (**H**) Representative fluorescence images of 2-NBDG-labeled glucose uptake in MIN6 cells after treatments with STZ and/or TAT-PBX1, scale bar = 200 μm (*n* = 3). (**I**) Quantification of relative fluorescence intensity from the 2-NBDG glucose uptake assay (*n* = 3). Statistical analyses were performed using Levene’s test for homogeneity of variance. Data meeting homogeneity of variance assumptions were analyzed using one-way ANOVA with the Holm–Šídák multiple comparisons test, while nonhomoscedastic data were evaluated using Kruskal–Wallis test with Dunn’s multiple comparisons test. a: STZ vs. Control, *p* < 0.05; b: TAT-PBX1 vs. STZ, *p* < 0.05; c: TAT-PBX1 + STZ vs. STZ, *p* < 0.05.

To investigate whether the therapeutic effects of TAT-PBX1 operate through restoration of insulin signaling and consequent recovery of glucose metabolism and secretory function, we examined insulin signaling components (IRS1, PI3K, PDK1, Akt and downstream GSK3β phosphorylation) and measured glucose uptake using 2-NBDG. STZ (1 mmol/L) significantly decreased IRS1 levels and the ratios of p-PI3K p85/PI3K p85, p-PDK1/PDK1, p-Akt/Akt and p-GSK3β/GSK3β compared with control, indicating suppression of insulin signaling (Figure 3A–G). TAT-PBX1 co-treatment significantly restored these phosphorylation markers; TAT-PBX1 alone also increased these indices in the absence of STZ, suggesting that TAT-PBX1 can enhance signaling competence (Figure 3B–G). Functionally, 2-NBDG fluorescence was reduced in the STZ group (*p* < 0.05 vs. control) and was significantly increased by TAT-PBX1 treatment (*p* < 0.05 vs. STZ); TAT-PBX1 alone did not differ from control, indicating no significant change in basal glucose uptake under these conditions (Figure 3H,I).

**Figure 4:**
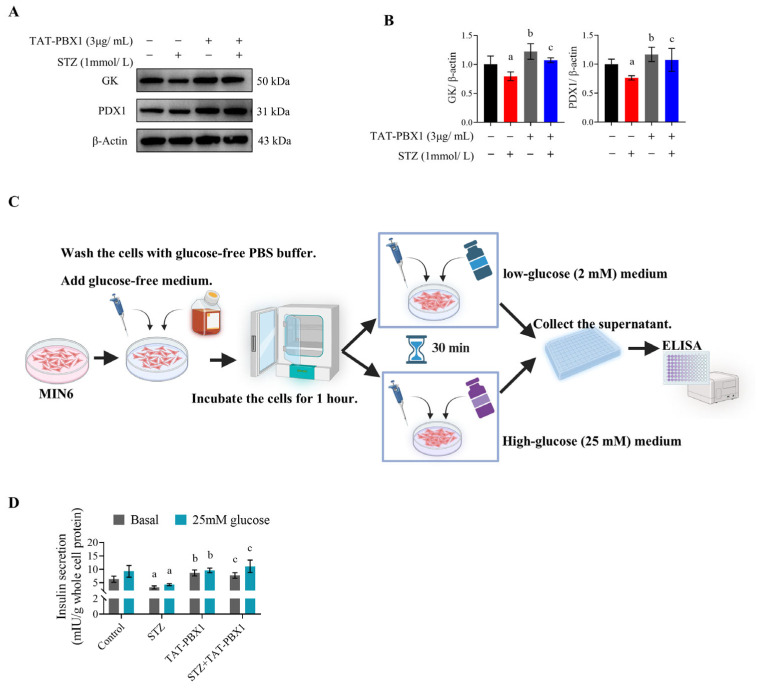
TAT-PBX1 restores GSIS in MIN6 cells. (**A**) Representative Western blot bands of GK and PDX1 protein expression after treatments with STZ and/or TAT-PBX1 (*n* = 3). (**B**) Quantitative analysis of GK and PDX1 protein expression (*n* = 3). (**C**) Schematic diagram of the GSIS assay. Briefly, after 24 h of treatment with STZ and/or TAT-PBX1, cells were washed once with PBS and incubated in glucose-free DMEM for 1 h. Basal secretion was collected after 30 min incubation with 2 mM glucose, followed by collection of stimulated secretion after 30 min incubation with 25 mM glucose. Insulin concentrations were measured by ELISA. (**D**) ELISA measurement of insulin secretion under basal (2 mM) and stimulated (25 mM) glucose conditions, normalized to total protein content of each group. Statistical analyses were performed using Levene’s test for homogeneity of variance. Data meeting homogeneity of variance assumptions were analyzed using one-way ANOVA with the Holm–Šídák multiple comparisons test, while nonhomoscedastic data were evaluated using the Kruskal–Wallis test with Dunn’s multiple comparisons test. For GSIS, data were analyzed using two-way repeated-measures ANOVA (factors: treatment group and glucose concentration) with Šídák multiple comparisons to assess within-group (25 mM vs. 2 mM) and between-group differences at each glucose level. a: STZ vs. Control, *p* < 0.05; b: TAT-PBX1 vs. STZ, *p* < 0.05; c: TAT-PBX1 + STZ vs. STZ, *p* < 0.05. Created in BioRender. Meng, X. (2025) https://BioRender.com/a94o930.

To evaluate effects on insulin secretion, PDX1 and GK protein levels were measured and GSIS assays were performed. STZ markedly downregulated PDX1 and GK expression; co-treatment with TAT-PBX1 reversed these decreases, restoring PDX1 and GK to levels approaching control (Figure 4A,B). In GSIS assays, the STZ group exhibited reduced basal (low-glucose) insulin release and did not show a significant increase in insulin secretion when glucose was raised from 2 mM to 25 mM, indicating loss of GSIS coupling (Figure 4C,D). By contrast, Control, TAT-PBX1 and STZ + TAT-PBX1 groups showed a significant within-group increase in insulin secretion under high-glucose stimulation, indicating restored GSIS. These data support that TAT-PBX1 restores insulin signaling and the PDX1–GK axis, thereby rescuing STZ-impaired insulin secretion in MIN6 cells.

### 2.4. Therapeutic Evaluation of TAT-PBX1 in an STZ-Induced Diabetic Mouse Model

We next evaluated TAT-PBX1 in an STZ-induced mouse model of diabetes. Model establishment was confirmed by sustained fasting hyperglycemia and reduced fasting insulin, with impaired glucose tolerance on intraperitoneal glucose tolerance test (IPGTT) further supporting β-cell functional impairment (Supplementary Figure S2B–F). STZ-injected mice showed impaired glucose tolerance with a significantly increased blood glucose AUC and elevated fasting blood glucose (FBG) compared with controls, meeting the predefined criteria for diabetes and eligibility for therapeutic testing. After randomization, mice were assigned to Control (saline), DM (saline), DM + Dorzagliatin (i.g. 10 mg/kg) and DM + TAT-PBX1 (i.p. 5 mg/kg) groups and dosed every other day for 8 weeks with periodic metabolic monitoring.

**Figure 5:**
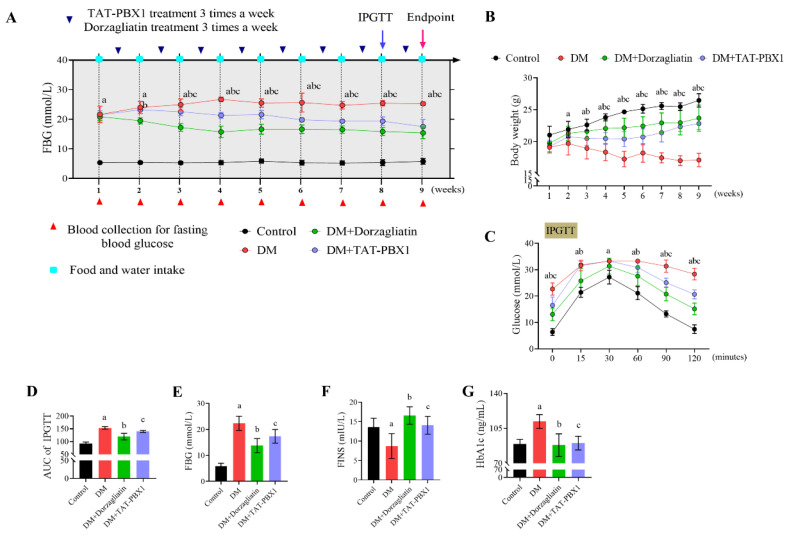
Therapeutic effect of TAT-PBX1 on STZ-induced diabetic mice. (**A**) Fasting blood glucose (FBG) changes in STZ-induced diabetic mice treated with Dorzagliatin and TAT-PBX1 (3 times per week, every other day) (*n* = 6). (**B**) Body weight changes in STZ-induced diabetic mice during Dorzagliatin and TAT-PBX1 treatment (*n* = 6). (**C**) Intraperitoneal glucose tolerance test (IPGTT) curves (*n* = 6). (**D**) Area under the IPGTT curves (*n* = 6). (**E**) FBG levels at week 9 (*n* = 6). (**F**) Fasting serum insulin (FINS) concentrations at week 9 (*n* = 6). (**G**) Glycated hemoglobin (HbA1c) levels at week 9 (*n* = 6). Time-course data (**A**–**C**) were analyzed using two-way repeated-measures ANOVA with factors time and group. Single-time-point endpoints (**D**–**G**) were analyzed using ordinary one-way ANOVA with Holm–Šídák multiple comparisons or the Kruskal–Wallis test with Dunn’s multiple comparisons, as appropriate. a: DM vs. Control, *p* < 0.05; b: DM + Dorzagliatin vs. DM, *p* < 0.05; c: DM + TAT-PBX1 vs. DM, *p* < 0.05.

At treatment baseline, the Control group had lower fasting glucose and higher body weight than the other groups (Figure 5A,B). Importantly, baseline FBG and body weight did not differ among the diabetic groups prior to treatment, indicating balanced randomization. Dorzagliatin reduced fasting glucose relative to the DM group from week 2, whereas TAT-PBX1 showed a significant glucose-lowering effect beginning at week 4 that persisted through study end, indicating earlier onset for Dorzagliatin and later but sustained efficacy for TAT-PBX1 (Figure 5A). Body weight was higher in Control at baseline; from week 3 Dorzagliatin increased weight relative to DM, and from week 4 TAT-PBX1 also increased weight relative to DM and maintained it to study end (Figure 5B). Food and water intake were elevated in DM, DM + Dorzagliatin and DM+TAT-PBX1 groups from week 1 versus Control (Supplementary Figure S3A,B). With treatment, food intake decreased in the DM + Dorzagliatin and DM+TAT-PBX1 groups from week 2, stabilizing by week 3 and remaining lower than DM; by week 5 water intake was significantly higher in DM than in treated groups, indicating symptomatic improvement with both interventions (Supplementary Figure S3A,B).

Terminal metabolic assessment showed that IPGTT AUC was significantly higher in DM mice than in Control, DM + Dorzagliatin and DM + TAT-PBX1 groups, indicating impairment of glucose tolerance and glucose clearance in untreated DM mice (Figure 5C,D); both Dorzagliatin and TAT-PBX1 improved these parameters. Endpoint biochemistry revealed elevated fasting glucose and HbA1c and reduced fasting insulin in DM mice compared with the other groups (Figure 5E–G), consistent with β-cell secretory dysfunction; maintenance or partial recovery of fasting insulin in Dorzagliatin and TAT-PBX1 groups supports preservation or partial restoration of β-cell function. Overall, TAT-PBX1 significantly ameliorated hyperglycemia and diabetes-related phenotypes in the STZ model and improved glucose tolerance, with sustained efficacy observed through the study endpoint.

### 2.5. TAT-PBX1 Improves Islet Pathology and Reduces Apoptosis In Vivo

**Figure 6:**
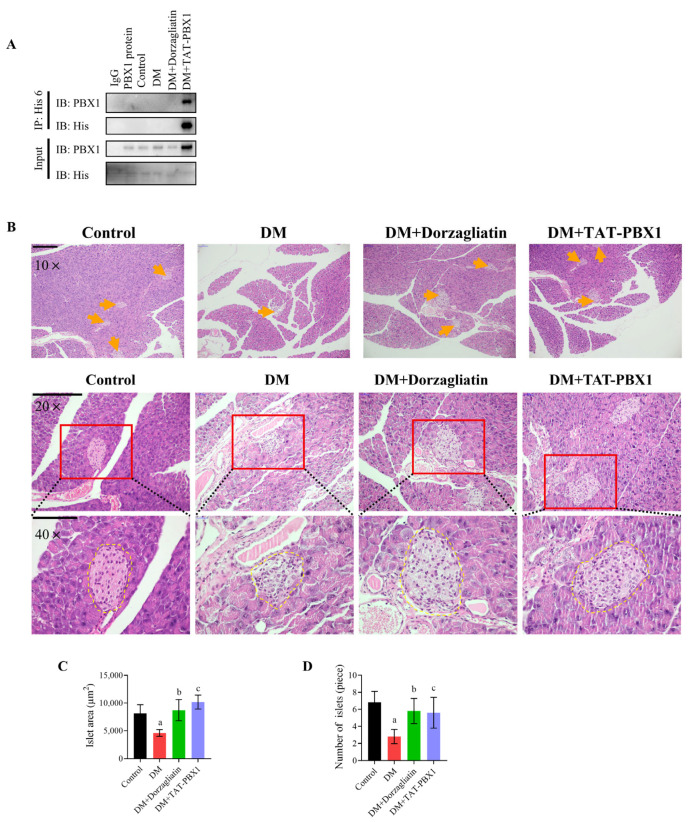
TAT-PBX1 improves islet pathology in STZ-induced diabetic mice. (**A**) Detection of His6-tagged TAT-PBX1 in pancreatic tissue lysates by anti-His immunoprecipitation (IP), with IgG as a negative control. Input indicates total lysate prior to IP. (**B**) Representative H&E-stained pancreatic tissue sections from each group. Scale bars: 10×, 200 μm; 20×, 200 μm; 40×, 100 μm. Yellow arrows indicate pancreatic islets. (**C**) Quantification of islet area (*n* = 3). (**D**) Quantification of islet number (*n* = 3). Statistical analyses were performed using Levene’s test for homogeneity of variance. Data meeting homogeneity of variance assumptions were analyzed using one-way ANOVA with the Holm–Šídák multiple comparisons test, while nonhomoscedastic data were evaluated using the Kruskal–Wallis test with Dunn’s multiple comparisons test. a: DM vs. Control, *p* < 0.05; b: DM + Dorzagliatin vs. DM, *p* < 0.05; c: DM + TAT-PBX1 vs. DM, *p* < 0.05.

At the end of the 8-week dosing period, IP assays detected TAT-PBX1 in pancreatic tissue lysates from the DM + TAT-PBX1 group (Figure 6A). The His-tagged signal was readily detectable in the anti-His pull-down, while no corresponding band was observed in the IgG control, and the input lane confirmed the presence of the target protein in the lysate, supporting pancreatic tissue exposure of the systemically administered fusion protein under our experimental conditions. Histological analysis revealed that STZ-treated DM mice exhibited marked islet atrophy and structural disorganization, whereas treatment with Dorzagliatin or TAT-PBX1 improved islet size, morphology and boundary integrity (Figure 6B). Quantitative analysis showed that both mean islet area and islet number were significantly reduced in DM mice versus controls; Dorzagliatin or TAT-PBX1 treatment significantly increased islet area and number relative to DM, partially or nearly restoring these metrics toward control levels (Figure 6C,D).

**Figure 7:**
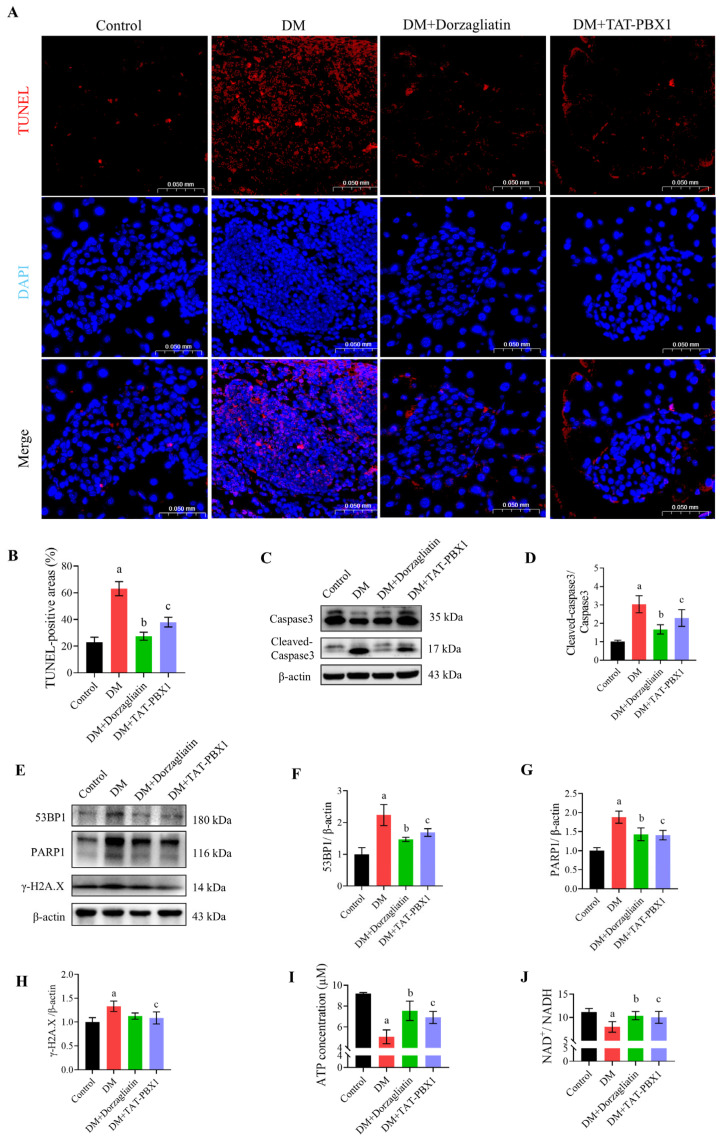
TAT-PBX1 reduces islet-associated apoptosis and stress-related markers in STZ-induced diabetic mice. (**A**) TUNEL staining of islets (scale bar = 0.05 mm, *n* = 3). (**B**) Quantification of TUNEL-positive areas (*n* = 3). (**C**) Representative Western blot bands of Caspase 3 and cleaved Caspase 3. (**D**) Ratio of cleaved Caspase 3 to Caspase 3 (*n* = 3). (**E**) Representative Western blot bands of 53BP1, PARP1, and γ-H2A.X. (**F**) Quantitative analysis of 53BP1 protein expression (*n* = 3). (**G**) Quantitative analysis of PARP1 protein expression (*n* = 3). (**H**) Quantitative analysis of γ-H2A.X protein expression (*n* = 3). (**I**) ATP concentrations in pancreatic tissues from each group (*n* = 3). (**J**) NAD^+^/NADH ratios in pancreatic tissues (*n* = 3). Statistical analyses were performed using Levene’s test for homogeneity of variance. Data meeting homogeneity of variance assumptions were analyzed using one-way ANOVA with the Holm–Šídák multiple comparisons test, while nonhomoscedastic data were evaluated using the Kruskal–Wallis test with Dunn’s multiple comparisons test. a: DM vs. Control, *p* < 0.05; b: DM + Dorzagliatin vs. DM, *p* < 0.05; c: DM + TAT-PBX1 vs. DM, *p* < 0.05.

TUNEL staining corroborated these findings: the DM group had markedly increased pancreatic cell apoptosis, which was significantly reduced after TAT-PBX1 or Dorzagliatin treatment (Figure 7A,B). Western blot analysis demonstrated an elevated cleaved caspase-3/caspase-3 ratio in DM pancreas that was reduced by both treatments (Figure 7C,D). Additionally, pancreatic tissue from DM mice showed increases in DNA damage markers (53BP1, γ-H2A.X) and PARP1; TAT-PBX1 markedly attenuated these changes toward control levels (Figure 7E–H). Dorzagliatin reduced 53BP1 and PARP1 but did not significantly affect γ-H2A.X. ATP content and the NAD^+^/NADH ratio were decreased in DM pancreatic tissue, and both were restored by Dorzagliatin or TAT-PBX1 (Figure 7I,J). Collectively, these data indicate that intraperitoneal TAT-PBX1 is detectable and enriched in pancreatic tissue and mitigates STZ-induced islet atrophy, DNA damage and apoptosis, thus contributing to the preservation of islet cell viability.

### 2.6. TAT-PBX1 Restores Insulin Signaling and Increases β-Cell Proliferative Markers In Vivo

**Figure 8:**
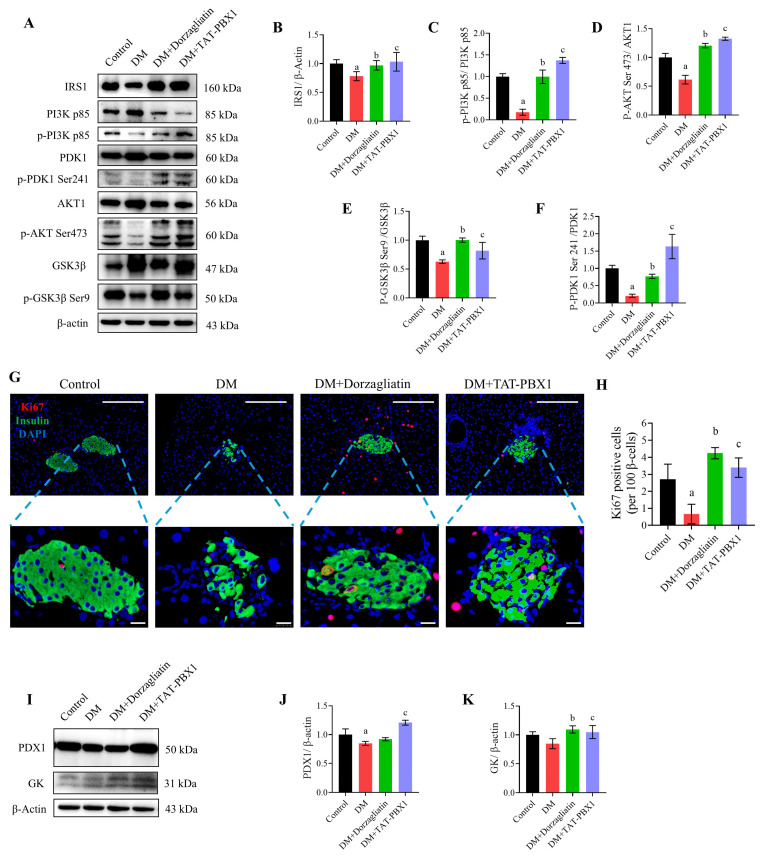
TAT-PBX1 restores insulin signaling and promotes β-cell proliferation. (**A**) Representative Western blot bands of IRS1, PI3K p85, p-PI3K p85, p-Akt S473, Akt, p-GSK3β S9, GSK3β, PDK1, and p-PDK1 S241 protein expression after treatments with STZ and/or TAT-PBX1 (*n* = 3). (**B**) Quantitative analysis of IRS1 protein expression (*n* = 3). (**C**) Ratio of p-PI3K p85 to PI3K p85. (**D**) Ratio of p-Akt S473 to Akt. (**E**) Ratio of p-GSK3β S9 to GSK3β. (**F**) Ratio of p-PDK1 S241 to PDK1. (**G**) Representative images of insulin (green) and Ki67 (red) double immunofluorescence staining in islets, scale bar = 200 μm, enlarged view scale bar = 20 μm. (**H**) Quantification of Ki67^+^ insulin^+^ cells (Ki67-positive β cells) in islets (*n* = 3). (**I**) Representative Western blot bands of GK and PDX1 protein expression in pancreatic tissue. (**J**) Quantitative analysis of GK protein expression (*n* = 3). (**K**) Quantitative analysis of PDX1 protein expression (*n* = 3). Statistical analyses were performed using Levene’s test for homogeneity of variance. Data meeting homogeneity of variance assumptions were analyzed using one-way ANOVA with the Holm–Šídák multiple comparisons test, while nonhomoscedastic data were evaluated using the Kruskal–Wallis test with Dunn’s multiple comparisons test. a: DM vs. Control, *p* < 0.05; b: DM + Dorzagliatin vs. DM, *p* < 0.05; c: DM + TAT-PBX1 vs. DM, *p* < 0.05.

Given TAT-PBX1’s effects on DNA damage reduction and energy homeostasis, we assessed its impact on insulin signaling, β-cell proliferation and the PDX1/GK axis in pancreatic tissue from STZ-induced diabetic mice. Western blot analysis showed that STZ-induced DM pancreas had reduced IRS1 protein and decreased ratios of p-PI3K p85/PI3K, p-PDK1 (Ser241)/PDK1, p-Akt (Ser473)/Akt and p-GSK3β (Ser9)/GSK3β compared with control, indicating suppression of this insulin-responsive signaling cascade (Figure 8A–F). Dorzagliatin or TAT-PBX1 treatment significantly restored these phosphorylation markers, increasing them toward control levels and consistent with partial functional recovery of the pathway. Immunofluorescence double-staining revealed a lower proportion of Ki67-positive β cells in DM mice versus controls, while both Dorzagliatin and TAT-PBX1 significantly increased Ki67 positivity within insulin-positive cells, suggesting enhanced β-cell proliferative marker expression under diabetic stress (Figure 8G,H). Consistently, PDX1 and GK protein expression, which were downregulated in DM pancreas, were partially restored toward control levels by both treatments (Figure 8I–K). Taken together, these findings indicate that TAT-PBX1 promotes coordinated recovery of insulin signaling and the PDX1/GK axis and is associated with increased Ki67 positivity in β cells, supporting improved β-cell functional status in the STZ-injury model.

## 3. Discussion

T1DM is typically caused by autoimmune–mediated destruction of pancreatic β cells, resulting in absolute insulin deficiency, whereas T2DM is initiated by peripheral insulin resistance accompanied by progressive β-cell dysfunction and loss, culminating in relative or absolute insulin deficiency [26]. Although the initiating etiologies differ, β-cell loss and secretory dysfunction are key determinants of hyperglycemia in both diseases [27]. Reliance on exogenous insulin alleviates hyperglycemia but does not reverse β-cell injury; moreover, chronic hyperglycemia and metabolic stress further exacerbate β-cell dysfunction [28]. Thus, beyond glycemic control, strategies that preserve and restore β-cell survival and function hold promise for durable metabolic benefit. Our results indicate that TAT-PBX1 restores STZ-induced β-cell dysfunction by two complementary mechanisms. On the one hand, it reduces STZ-induced DNA damage markers in pancreatic tissue, helping to preserve cellular integrity and metabolic competence. On the other hand, it supports restoration of insulin signaling to regulate GK activity, thereby restoring glucose sensing and dynamic insulin secretion. These findings support a potential role for PBX1 in the maintenance of β-cell function and suggest that augmenting PBX1 activity helps reinforce β-cell identity under diabetic stress.

Unlike oral agents that act via cell-surface receptors or metabolic enzymes, this cell-penetrating fusion protein may engage PBX1-dependent intracellular regulatory programs to bolster β-cell resilience and function. Conventional antihyperglycemic agents like metformin, sulfonylureas and DPP-4 inhibitors principally act by lowering blood glucose or improving peripheral insulin sensitivity, but they do not reverse β-cell failure [29,30,31]. Dorzagliatin can directly stimulate GK and enhance insulin secretion, but its efficacy may depend on residual β-cell function and the durability of its effects requires further study [32]. In our experimental model, TAT-PBX1 not only produced glucose-lowering effects but was also associated with improvements in cell survival, bioenergetic indices, and restoration of insulin-signaling molecules, suggesting it may promote both β-cell survival and metabolic recovery. Mechanistically, Dorzagliatin directly activates GK, whereas PBX1 delivery appears to act upstream by improving β-cell energetics and insulin-responsive signaling, thereby facilitating recovery of the PDX1/GK axis and secretory function.

DNA damage and the resultant β-cell apoptosis are recognized contributors to islet failure. Elevated markers of DNA damage have been detected in peripheral blood from T1DM patients, indicating a systemic DNA damage burden under metabolic stress [33,34]. In T2DM, chronic glucolipotoxicity and ROS accumulation similarly increase β-cell DNA damage and drive functional decline [35]. Thus, DNA damage appears to be a shared pathological driver of β-cell apoptosis across diabetes types. STZ induces DNA alkylation and oxidative stress, activating DNA-repair pathways and triggering β-cell toxicity [36]. Beyond direct genomic injury, DNA damage can worsen cellular dysfunction via PARP1-mediated NAD^+^ consumption and consequent energetic collapse. Preclinical studies show that NAD^+^ supplementation or PARP inhibition can mitigate STZ-induced declines in islet NAD^+^ and ATP and reduce β-cell apoptosis [37,38,39]. However, clinical meta-analyses have reported associations between PARP inhibitors and increased hyperglycemia risk, suggesting PARP inhibition may perturb insulin secretion or glucose homeostasis [40]. Likewise, long-term high-dose NAD^+^ precursor supplementation might alter epigenetic regulation, potentially impacting β-cell function or systemic insulin sensitivity [41]. Therefore, targeting DNA damage alone or simply boosting NAD^+^ pools may be insufficient for durable β-cell protection. This highlights the need for interventions that mitigate genotoxic stress without compromising metabolic signaling. Notably, TAT-PBX1 achieves this dual benefit by preserving NAD^+^/ATP levels while reducing DNA damage, potentially avoiding the metabolic side effects observed with PARP inhibition. Notably, STZ and TAT-PBX1 exert different effects on PARP1 in MIN6 cells versus mouse pancreatic tissue. The contrasting effects of STZ on PARP1 expression in vitro versus in vivo likely reflect the different contexts of acute cytotoxicity and chronic diabetic stress. Acute, severe DNA damage in MIN6 cells may lead to PARP1 hyperactivation and cleavage, whereas in the chronic in vivo setting, sustained low-level damage might trigger compensatory upregulation of DNA repair components, including PARP1. In both scenarios, TAT-PBX1 effectively normalizes PARP1-related pathology, underscoring its ability to stabilize the DNA damage response network under diverse stress conditions, but further investigation is needed. Our results show that TAT-PBX1 increases ATP supply, thereby reducing both the cumulative consequences of DNA damage and apoptosis driven by energetic collapse, which establishes a cellular basis for recovery of insulin secretion.

The restoration of β-cell secretory capacity is a cornerstone of diabetes management and potential functional improvement. Our data position TAT-PBX1 as a key mediator of this process. It reactivates the proximal insulin signaling pathway (IRS1/PI3K/Akt), which in turn is associated with increased PDX1 protein abundance [42]. The upregulation of PDX1 drives the expression of both GK, the glucose sensor, and insulin itself. This coordinated molecular reinstatement ultimately closes the functional loop: enhanced glucose metabolism elevates the ATP/ADP ratio, triggering membrane depolarization, calcium influx, and the recovery of GSIS. Small-molecule GK activators improve glucose sensitivity, enhance GSIS, and lower HbA1c, indicating that direct GK activation can improve glucose metabolism; however, these agents rely on residual β-cell signaling capacity for efficacy [43]. In addition, according to recent clinical and animal experiments, the use of GKA should be carefully considered because of side effects such as increased blood lipids, increased uric acid, and liver fat accumulation [44,45]. These findings have been substantiated in recent studies: a meta-analysis of GKA trials reported higher odds of hyperlipidemia and hyperuricemia, and a preclinical study found that chronic GKA exposure induced significant hepatic fat accumulation [46,47]. Given these reported liabilities, Dorzagliatin served here as a GK-axis comparator in the STZ injury context rather than as an endorsement of chronic GKA use in insulin-deficient diabetes. Our data further indicate that Dorzagliatin and TAT-PBX1 can yield broadly similar regeneration-associated improvements; however, these endpoints should be interpreted as an integrated net-recovery phenotype that may reflect reduced apoptosis, preserved tissue architecture, improved bioenergetic status, and partial restoration of β-cell functional identity, rather than necessarily true β-cell mass expansion. Such phenotypic convergence does not imply mechanistic equivalence. Dorzagliatin acts at the GK node to enhance glucose sensing and ATP generation, whereas TAT-PBX1 restores a more upstream milieu—stabilizing cellular energetics and insulin-responsive signaling, thereby secondarily supporting the PDX1–GK axis and GSIS competence. Importantly, TAT-PBX1 increased the fraction of Ki67-positive β cells, suggesting increased β-cell proliferative signaling or cell-cycle entry under diabetic stress, which may derive from restored Akt signaling by promoting cell-cycle regulators, limiting FOXO1 nuclear activity, and improving energy and substrate availability for biosynthesis. This is notable given that adult β cells have very limited proliferative capacity under normal conditions, indicating that TAT-PBX1 may help overcome intrinsic restraints on β-cell replication. Prior studies implicate PBX1 in AKT/GSK3β-regulated cell proliferation, consistent with our observations [23]. Thus, TAT-PBX1 may both restore β-cell secretory function and create a signal- and energy-favorable milieu supporting β-cell mass maintenance and regenerative potential.

Several limitations merit comment. We used an STZ-induced insulin-deficient β-cell injury model and male mice only; validation across sexes and in autoimmune and insulin-resistance–based models will strengthen generalizability. Because TAT-PBX1 was delivered systemically, biodistribution, potential off-target exposure, and long-term safety (including immunogenicity) warrant further evaluation. In addition, direct PBX1 transcriptional targets and downstream mitochondrial programs remain to be delineated in future mechanistic studies.

## 4. Materials and Methods

### 4.1. Construction, Purification, and Endotoxin Removal of TAT-PBX1

The construction of TAT-PBX1 was performed based on previously described procedures [48]. Briefly, the full-length PBX1 coding sequence was amplified from the plasmid pLVX-PBX1-IRES-mCherry and subcloned into the pTAT-HA vector to generate the expression plasmid pTAT-HA-PBX1. The recombinant plasmid was transformed into *E. coli* BL21(DE3) and cultured in LB medium containing the appropriate antibiotic until reaching the exponential phase, followed by induction at 22 °C for 20 h. Bacterial pellets were collected, lysed by sonication, and centrifuged to separate the soluble and insoluble fractions. TAT-PBX1 fusion protein was purified using a Ni^2+^-NTA affinity column and subsequently refolded through a denaturation–renaturation procedure. The refolded protein was dialyzed into PBS, concentrated, and quantified using a BCA assay. Endotoxin was removed using ultrafiltration with a 100 kDa molecular weight-cutoff membrane, allowing separation of endotoxin aggregates (>100 kDa) from proteins and buffer components. Subsequently, the purified protein was further subjected to endotoxin removal using a specialized endotoxin-removing resin. The protein solution was then passed through a 0.22-μm endotoxin-free sterile filter prior to use in in vitro and in vivo experiments. Protein purity and identity were confirmed by SDS-PAGE and Western blotting.

### 4.2. Cell Culture

The mouse pancreatic β-cell line MIN6 was obtained from the Cell Bank of the Chinese Academy of Sciences (Shanghai, China). Cells were maintained in high-glucose Dulbecco’s Modified Eagle Medium (DMEM, 11965118, Gibco, Grand Island, NY, USA) supplemented with 10% (*v*/*v*) fetal bovine serum (FND500, ExCell, Suzhou, China) and 100 IU/mL penicillin–streptomycin (SC118-01, Seven, Beijing, China). Cultures were incubated at 37 °C in a humidified atmosphere containing 5% CO_2_. Cells were passaged at a 1:2 ratio once they reached approximately 80% confluence. Cells used for experiments were recovered from cryopreservation and passaged twice before use.

### 4.3. STZ-Induced MIN6 Cell Injury Model

To establish a stable β-cell injury model, MIN6 cells were seeded into 96-well plates at 1.5 × 10^4^ cells/well and allowed to attach for 24 h. STZ was freshly prepared on ice and used within 5 min, and was added to achieve final concentrations of 0.1, 0.5, 1, 1.5, 2, 2.5, 5, and 10 mmol/L. After 24 h of incubation with STZ, cell viability was assessed using the CCK-8 assay. STZ decreased MIN6 viability in a dose-dependent manner, and ~1 mmol/L reduced viability to approximately 60% (Supplementary Figure S1), a level generally considered indicative of moderate injury without excessive cell death. Therefore, 1 mmol/L STZ was selected for subsequent experiments.

### 4.4. CCK-8 Cell Viability Assay

MIN6 cells were seeded in 96-well plates at 5 × 10^3^ cells/well in 100 μL complete medium. After treatment, 10 μL of CCK-8 reagent (SC119-02, Seven, Beijing, China) was added to each well and incubated for 1 h at 37 °C in the dark. Absorbance was measured at 450 nm using a microplate reader.

### 4.5. Annexin V/PI Flow Cytometry

Apoptosis in MIN6 cells was evaluated using the Annexin V-FITC/PI apoptosis detection kit (556547, BD Biosciences, San Jose, CA, USA). Treated cells were harvested by gentle trypsinization, washed twice with cold PBS, and resuspended in 1× binding buffer at ~1 × 10^6^ cells/100 μL. Annexin V-FITC and PI were added, and cells were incubated at room temperature for 15 min in the dark. After adding 400 μL of binding buffer, samples were analyzed immediately by flow cytometry, collecting at least 1 × 10^4^ events per sample. Data were analyzed with FlowJo v10.8.1 (BD Biosciences, USA).

### 4.6. 2-NBDG Glucose Uptake Assay

Following treatment, MIN6 cells were starved in glucose-free PBS for 30 min. Cells were then incubated with 50 μM 2-NBDG (S8914, Selleck, Shanghai, China) at 37 °C for 45 min in the dark. After incubation, cells were washed three times with ice-cold PBS. Fluorescence was measured using a microplate reader (Cytation 3, BioTek, Shoreline, WA, USA; Ex: 465 nm, Em: 540 nm).

### 4.7. GSIS

After 24 h of treatment with STZ and/or TAT-PBX1, cells were washed once with PBS and incubated for 1 h in glucose-free DMEM (11966025, Gibco, USA). Cells were then incubated with 2 mM glucose for 30 min to collect baseline secretion, followed by 25 mM glucose for another 30 min to collect stimulated secretion. Supernatants were centrifuged at 12,000× *g* for 15 min and stored at −80 °C. Cell lysates were prepared by repeated freeze–thaw cycles and sonication, and total protein was quantified by BCA assay. Insulin concentration was measured using a mouse insulin Enzyme-linked immunosorbent assay (ELISA) kit and normalized to total cellular protein.

### 4.8. Animals and Experimental Design

#### 4.8.1. Experimental Animals

Eight-week-old male C57BL/6J mice (20–25 g) were purchased from Beijing Huafukang Biotechnology Co., Ltd. (Beijing, China). Experiments were performed in the animal facility of the School of Public Health, Jilin University, following approval by the Institutional Animal Care and Use Committee (Approval No. SY202511007). The study was performed at the School of Public Health, Jilin University (SYXK (Ji) 2021-0003) with oversight from the Institutional Animal Care and Use Committee of Jilin University. All procedures conformed to the GB2018-35892 Guidelines for Ethical Welfare Review of Laboratory Animals, and animal housing conditions met the requirements of GB14925. Mice were housed under controlled conditions (20–24 °C, 40–60% humidity, 12 h light/dark cycle, 15 air changes/h) with free access to standard chow and water. Animals were acclimatized for at least 7 days before experimentation.

#### 4.8.2. STZ-Induced Insulin-Deficient (T1DM-like) Mouse Model

STZ is a pancreatic β-cell–selective diabetogenic agent that induces β-cell injury and insulin deficiency, and it is widely used to establish experimental models of insulin-deficient diabetes. This multiple low-dose STZ regimen is a widely used model of insulin-deficient diabetes driven by β-cell injury; however, it does not fully recapitulate the autoimmune etiology of human T1DM. The protocol for establishing the STZ-induced insulin-deficient (T1DM-like) mouse model is shown in Supplementary Figure S2A. STZ (S0130, Sigma, St. Louis, MO, USA) was freshly dissolved in cold 0.1 M citrate buffer (pH 4.5) before each injection. Insulin-deficient (T1DM-like) diabetes was induced using a multiple low-dose regimen: 50 mg/kg STZ was administered intraperitoneally once daily for 5 consecutive days at 9:30 a.m. Control mice received an equivalent volume of citrate buffer. Mice were monitored daily for clinical signs. FBG was monitored for three consecutive days on days 17–19 (i.e., 5–7 days after the final injection). Mice with FBG ≥ 11.1 mmol/L for three consecutive days were considered diabetic. IPGTT was performed on day 20 to confirm β-cell dysfunction and model stability.

#### 4.8.3. Experimental Groups and Treatment

Successfully modeled STZ-induced insulin-deficient (T1DM-like) diabetic mice and their controls were randomized into four groups (*n* = 6 each group): Control (CON): saline i.p.; DM: diabetic mice receiving saline i.p.; DM + Dorzagliatin: oral gavage (i.g.) of dorzagliatin (10 mg/kg in saline); DM + TAT-PBX1: TAT-PBX1 administered i.p. at 5 mg/kg. Based on previous studies, the therapeutic dose of dorzagliatin was 10 mg/kg [49,50]. Dorzagliatin was administered by oral gavage (i.g.) as this is the standard and clinically relevant route for this GK activator. TAT-PBX1 was administered intraperitoneally (i.p.) to ensure efficient systemic delivery of the protein therapeutic, a common practice for preclinical evaluation of peptide and protein drugs. All treatments were administered every other day for 8 weeks (3 times per week for 8 weeks, 28 doses in total). Body weight, food intake, and water consumption were monitored throughout. After the final treatment, mice underwent IPGTT. Blood and tissues were collected for biochemical analyses and histology.

#### 4.8.4. IPGTT

After a 6 h fast, baseline blood glucose was measured (0 min). Glucose (2 g/kg, dissolved in PBS) was injected intraperitoneally, and blood glucose was measured at 15, 30, 60, 90, and 120 min. The area under the curve (AUC) was calculated.

### 4.9. ELISA

After 24 h of STZ and/or TAT-PBX1 treatment, the culture supernatant of MIN6 cells was collected. After the mouse experiment was completed, blood was collected and centrifuged at 3000 rpm for 15 min at 4 °C, the serum was separated, and stored at −80 °C. Both cell supernatant and serum samples were tested for insulin (JM-02862, Jiangsu Jingmei Biotechnology Co., Ltd., Yancheng, China) and glycated hemoglobin (HbA1c, JM-02794, Jiangsu Jingmei Biotechnology Co., Ltd., China) using the corresponding ELISA kits, following the manufacturer’s instructions. For each group of cell supernatants, three replicate wells were set. Absorbance at 450 nm was measured using a microplate reader, and the insulin concentration secreted by each group of cells was calculated according to the standard curve.

### 4.10. ATP Measurement

ATP levels in MIN6 cells and pancreatic tissue were measured using a commercial ATP Assay Kit (S0026, Beyotime, Shanghai, China) following the manufacturer’s protocol. For MIN6 cell samples, cells were harvested by trypsinization and centrifuged at 1000 rpm for 5 min. The resulting cell pellet was washed twice with pre-chilled PBS, centrifuged, and the supernatant discarded after each wash. Subsequently, 100 μL of ATP lysis buffer was added per 10^6^ cells. The cell suspension was vortexed thoroughly and lysed on ice for 10 min, followed by centrifugation at 12,000 rpm at 4 °C for 5 min to obtain the clarified supernatant for ATP quantification. For pancreatic tissue samples, approximately 20 mg of mouse pancreas was collected, and 200 μL of ATP lysis buffer was added. The tissue was mechanically homogenized on ice using zirconium beads. After resting at room temperature for 3–5 min to eliminate background luminescence, the homogenate was centrifuged at 12,000 rpm at 4 °C for 5 min. The resulting supernatant was collected and used for subsequent ATP measurement according to the kit instructions.

### 4.11. NAD^+^/NADH Ratio

NAD^+^/NADH in MIN6 cells and mouse pancreatic tissue was measured using the NAD^+^/NADH Assay Kit (S0175, Beyotime, China) and the WST-8 method. For adherent MIN6 cells, approximately 1.0 × 10^6^ cells were collected, the medium was removed, and 200 μL of extraction buffer was added per 10^6^ cells for lysis; for pancreatic tissue, approximately 10–30 mg of tissue was taken and minced/homogenized in 200 μL of extraction buffer per 10 mg of tissue. All samples were centrifuged at 12,000× *g*, 4 °C, for 5–10 min, and the clarified supernatant was collected. Total NAD measurement: 20 μL of sample diluted with extraction buffer was added to a 96-well plate. Reaction mixture was added according to the kit instructions, incubated at 37 °C in the dark for 10 min, and absorbance was measured at 450 nm, with concentration calculated from the standard curve. NADH measurement: 50–100 μL of sample was placed in a centrifuge tube and heated at 60 °C in a water bath or PCR instrument for 30 min to decompose NAD^+^. If precipitation occurred, samples were centrifuged at 10,000× *g* to remove debris. The clarified supernatant was diluted, added to the reaction mixture, incubated, and absorbance at 450 nm was measured. Concentration was calculated using the NADH standard curve. NAD^+^ was calculated as NAD^+^ = NAD total − NADH.

### 4.12. Western Blot

Western blot analysis was performed according to published methods [51]. Briefly, for cell samples, after removing the culture medium, cells were washed three times with PBS containing 1% protease inhibitor (P1005, Beyotime, Shanghai, China) and collected by centrifugation. Cells were then placed on ice and lysed for 20 min in RIPA lysis buffer (SW104, Sevenbio, Beijing, China) supplemented with PMSF (P7626, Sigma, USA), protease inhibitor, and phosphatase inhibitor (SW107, Sevenbio, China). For mouse pancreatic tissue samples, tissue was ground in liquid nitrogen and lysed in ice-cold RIPA buffer for 20 min. During lysis, samples were gently vortexed and then centrifuged at 14,000× *g*, 4 °C, for 15 min, and the supernatant was collected for analysis. Protein concentration was measured using a BCA Protein Quantification Kit (ZJ101, Epizyme, Shanghai, China). Equal amounts of protein were separated by 8–12% SDS-PAGE and transferred to PVDF membranes (IPVH00010, Millipore, Billerica, MA, USA). Membranes were blocked at room temperature with 5% skim milk or 5% BSA for 1 h to prevent nonspecific binding. Membranes were then incubated overnight at 4 °C with primary antibodies: PBX1 (HA721303, dilution ratio 1:1000, Huabio, Hangzhou, China), Caspase 3 (AF6311, dilution ratio 1:1000, Affinity, Shanghai, China), Cleaved Caspase 3 (9661, dilution ratio 1:1000, CST, Danvers, MA, USA), GK (DF8549, dilution ratio 1:1000, Affinity, China), Pancreatic and Duodenal Homeobox 1 (PDX1, 20989-1-AP, dilution ratio 1:1000, Proteintech, Wuhan, China), p53-binding protein 1 (53BP1, ab36823, dilution ratio 1:1000, Abcam, Cambridge, UK), PARP1 (9532, dilution ratio 1:1000, CST, USA), γ-H2A.X (2577, dilution ratio 1:1000, CST, USA), Insulin Receptor Substrate 1 (IRS1, 17509-1-AP, dilution ratio 1:1000, Proteintech, China), Phosphoinositide 3-kinase regulatory subunit p85 (PI3K p85, T40115, dilution ratio 1:1000, Abmart, Shanghai, China), Phosphorylated PI3K p85 (p-PI3K p85, T40116, dilution ratio 1:1000, Abmart, China), AKT (9272, dilution ratio 1:1000, CST, USA), Phosphorylated AKT Ser473 (p-AKT S473, 9271, dilution ratio 1:1000, CST, USA), 3-Phosphoinositide-dependent protein kinase-1 (PDK1, F1103, dilution ratio 1:1000, Selleck, Houston, TX, USA), Phosphorylated PDK1 Ser241 (p-PDK1 S241, F0401, dilution ratio 1:1000, Selleck, Shanghai, China), GSK3β (F0142, dilution ratio 1:1000, Selleck, USA), Phosphorylated GSK3β Ser9 (p-GSK3β S9, F0299, dilution ratio 1:1000, Selleck, USA), β-Actin (PSH03-63, dilution ratio 1:1000, Huabio, China). The next day, membranes were washed three times with TBST and incubated with HRP-conjugated secondary antibodies at room temperature for 2 h. Protein bands were visualized using ECL substrate, and band intensity was semi-quantified using ImageJ software (version 1.8.0.172, National Institutes of Health, Bethesda, MD, USA).

### 4.13. Immunoprecipitation

To detect TAT-PBX1 protein in mouse pancreatic tissue, an Anti-His immunoprecipitation (IP) kit (IK-1015, Biolinkedin, Shanghai, China) was used following the manufacturer’s instructions. Briefly, approximately 50 mg of mouse pancreas was ground in liquid nitrogen and homogenized in ice-cold RIPA lysis buffer (supplemented with 1% PMSF and protease inhibitor cocktail) and lysed on ice for 30 min. Samples were centrifuged at 12,000× *g*, 4 °C, for 10 min, and the clarified lysate was collected and quantified using a BCA assay. A portion of lysate was kept as the Input control, and the remaining equal protein amounts were incubated with Anti-His magnetic beads at 4 °C with gentle rotation for 4 h (with IgG-conjugated beads as a negative control). After incubation, magnetic beads were separated using a magnetic stand, washed twice with ice-cold RIPA buffer, resuspended in 1× SDS loading buffer, and heated at 100 °C for 10 min to elute bound proteins. The eluted samples were denatured and analyzed by Western blot to detect His tag and PBX1 expression. Recombinant PBX1 protein was purchased from Proteintech (Ag12817).

### 4.14. H&E Staining

After mice were sacrificed, pancreatic tissue was immediately removed and washed with saline to remove blood. The tissue was fixed in 4% paraformaldehyde at room temperature for 48 h. After fixation, the tissue was dehydrated through graded ethanol, cleared in xylene, and embedded in paraffin. After dewaxing, sections were rehydrated sequentially with xylene, absolute ethanol, and 70% ethanol. Sections were stained with hematoxylin for 5–10 min, rinsed with running water, differentiated with 1% acid alcohol, and blued under running water for 10 min. They were then stained with eosin for 2 min, rinsed, dehydrated through graded ethanol, cleared in xylene, and mounted. Pancreatic morphology and islet structure were observed and imaged using a light microscope.

### 4.15. TUNEL Staining

Apoptosis of mouse pancreatic cells was assessed using a TUNEL kit (G1502, Servicebio, Wuhan, China). Pancreatic tissue was fixed in 4% paraformaldehyde for 48 h, paraffin-embedded, and sectioned into 3–4 μm slices. Sections were dewaxed twice in xylene, rehydrated through graded ethanol (100%, 95%, 90%, 80%, 70%), and washed twice with PBS. Sections were treated with proteinase K working solution at 37 °C for 15–30 min and washed twice with PBS. TUNEL reaction mixture was prepared from TdT enzyme and fluorescently labeled dUTP, and sections were incubated in a humidified, light-protected chamber at 37 °C for 60 min. After washing with PBS, DAPI staining was performed at room temperature in the dark for 10–20 min. Sections were mounted and observed under a fluorescence microscope to assess islet apoptosis.

### 4.16. Immunofluorescence Staining

Paraffin-embedded mouse pancreatic sections were dewaxed and rehydrated (xylene twice, 10 min each; graded ethanol 100%, 95%, 90%, 80%, 70%, 5 min each), rinsed in deionized water, and subjected to antigen retrieval in sodium citrate buffer (pH 6.0), heated to boiling, and maintained at 95 °C for approximately 15 min in a microwave or water bath, followed by natural cooling to approximately 25 °C and rinsing three times with PBS (5 min each). Sections were permeabilized with 0.1% Triton X-100 at room temperature for 15 min, followed by three PBS washes. Sections were blocked in 1% BSA solution in a humid chamber at 37 °C for 1 h to reduce nonspecific binding; then diluted primary antibodies anti-Ki67 (GB111499, dilution ratio 1:500, Servicebio, China) and anti-Insulin (GB11334, dilution ratio 1:100, Servicebio, China) prepared in 1% BSA were added and incubated overnight at 4 °C in the dark. The next day, sections were washed three times with PBS (5 min each), incubated with fluorescent secondary antibodies at room temperature in the dark for 1 h, washed three times, and mounted with antifade medium containing DAPI. Images were captured using a fluorescence microscope, with at least three random fields selected per section, and fluorescence intensity and colocalization were quantified using ImageJ after background subtraction.

### 4.17. Statistical Analysis

All results were analyzed using GraphPad Prism v8.0.2 and presented as mean ± SD. Normality was assessed using the Shapiro–Wilk test, and homogeneity of variance was assessed using Levene’s test. A two-tailed unpaired Student’s t-test was used for comparisons between two groups. Data meeting homogeneity of variance assumptions were analyzed using one-way ANOVA with the Holm-Šídák multiple comparisons test, while nonhomoscedastic data were evaluated using the Kruskal–Wallis test with Dunn’s multiple comparisons test. For repeated measurements/time-series data (e.g., body weight, FBG, food/water intake, IPGTT curves), two-way repeated-measures ANOVA was used to evaluate time, treatment group, and their interaction. A *p* value < 0.05 was considered statistically significant.

## 5. Conclusions

Taken together, our study provides proof-of-concept evidence that the cell-penetrating TAT-PBX1 fusion protein can mitigate STZ-associated β-cell injury and support functional recovery in diabetes models. Its proposed mechanism, which integrates cytoprotection, through mitigation of DNA damage and energetic collapse, with functional restoration via reactivation of insulin signaling and GSIS, and enhanced proliferative signaling, addresses the core pathologies of β-cell failure. This work provides compelling preclinical evidence for protein-based therapy that targets master regulatory transcription factors to support restoration of islet cell metabolism and function. Future investigations should prioritize evaluating the efficacy of TAT-PBX1 in autoimmune models, delineating its long-term safety profile, and further supporting its clinical translation.

## Data Availability

The original contributions presented in this study are included in the article and Supplementary Materials. Further inquiries can be directed to the corresponding author.

## Supplementary Materials

The following supporting information can be downloaded at: https://www.mdpi.com/article/10.3390/ph19010085/s1. Figure S1: Screening of STZ concentrations for cell experiments. Figure S2: Construction and validation of the STZ-induced insulin-deficient (T1DM-like) mouse model. Figure S3: Food and water intake in each group of mice.

## Abbreviations

53BP1: p53-binding protein 1; AUC: area under the curve; DMEM: Dulbecco’s Modified Eagle Medium; ELISA: Enzyme-linked immunosorbent assay; FBG: fasting blood glucose; FINS: fasting insulin; GK: glucokinase; GLUT2: Glucose Transporter 2; GSIS: glucose-stimulated insulin secretion; GSK3β: Glycogen synthase kinase-3 beta; HbA1c: glycated hemoglobin; IP: immunoprecipitation; IPGTT: intraperitoneal glucose tolerance test; IRS1: Insulin Receptor Substrate 1; PARP1: poly(ADP-ribose) polymerase 1; PBX1: Pre-B-cell leukemia homeobox 1; PDK1: 3-Phosphoinositide-dependent protein kinase-1; PDX1: Pancreatic and Duodenal Homeobox 1; PI3K p85: Phosphoinositide 3-kinase regulatory subunit p85; STZ: streptozotocin; T1DM: type 1 diabetes mellitus; T2DM: type 2 diabetes mellitus.
