# Supplementary figures and images for: TAT-PBX1 Reverses Hyperglycemia Through β-Cell Regeneration and Functional Restoration in an STZ-Induced Diabetic Model
Source: Pharmaceuticals (Basel). 2026 Jan 1;19(1):85. doi: 10.3390/ph19010085 (PMC12845013; doi:10.3390/ph19010085)

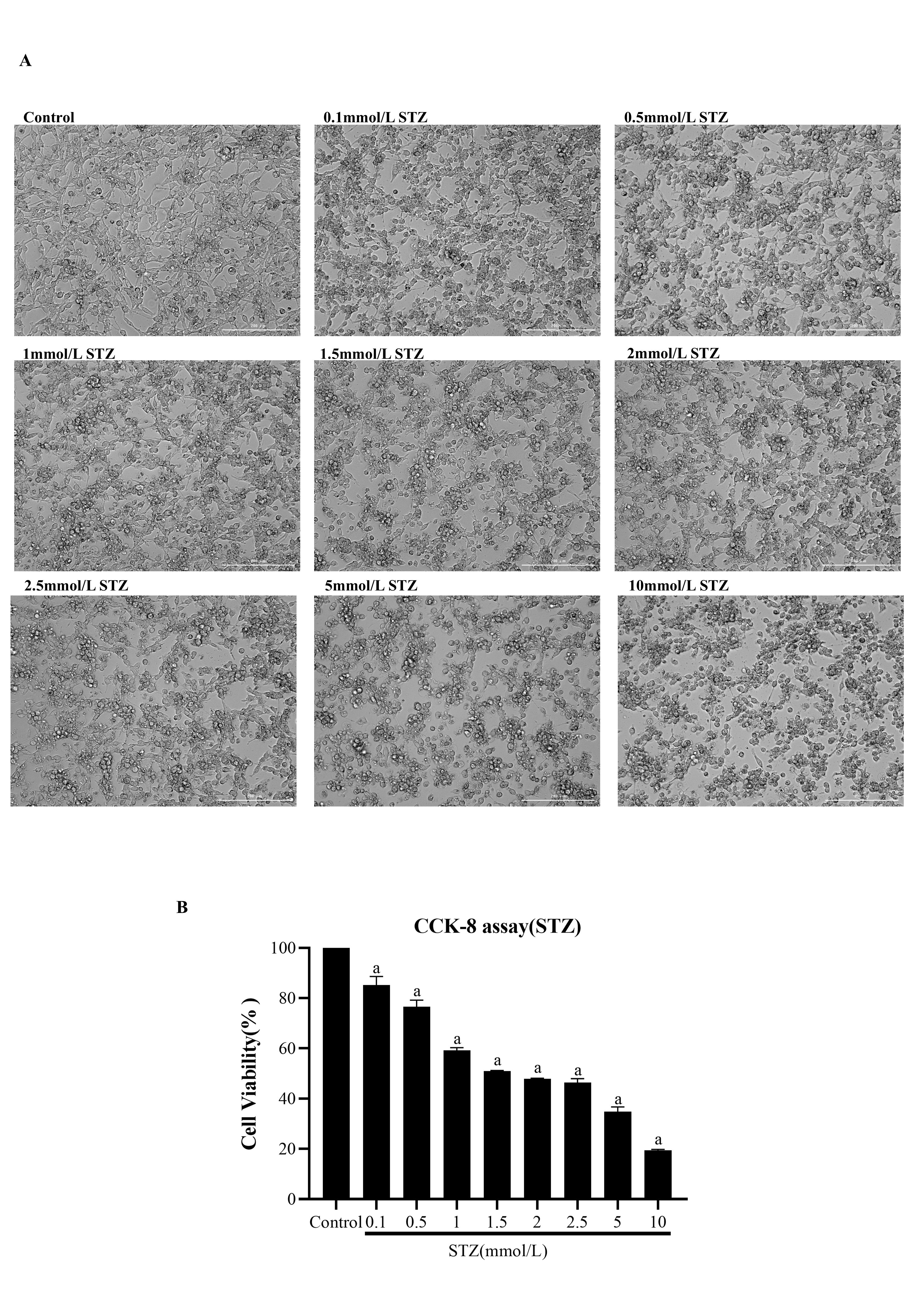

Supplement: Supplementary file 1 [file pharmaceuticals-19-00085-s001.zip › Figure S1.TIF]

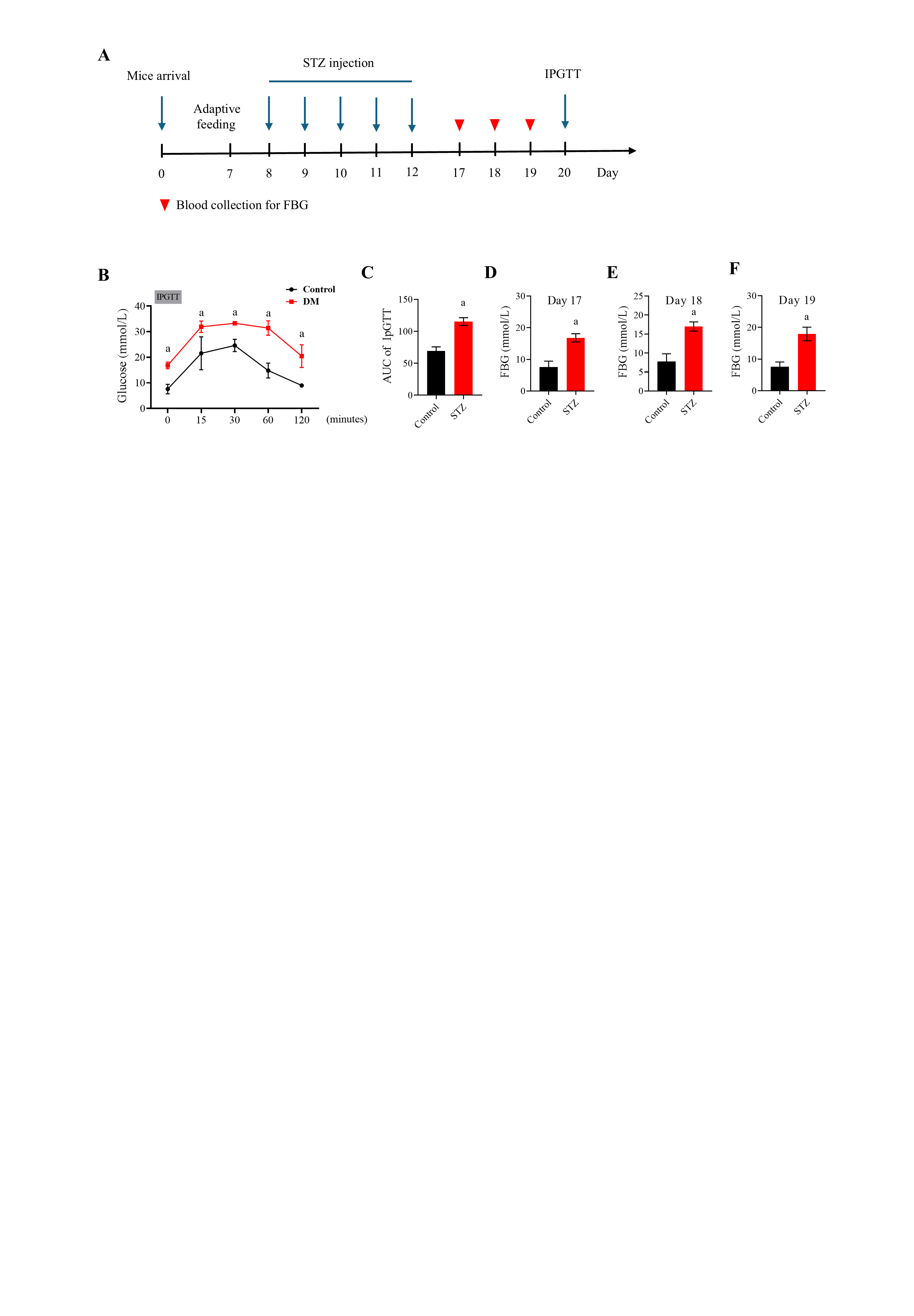

Supplement: Supplementary file 1 [file pharmaceuticals-19-00085-s001.zip › Figure S2.TIF]

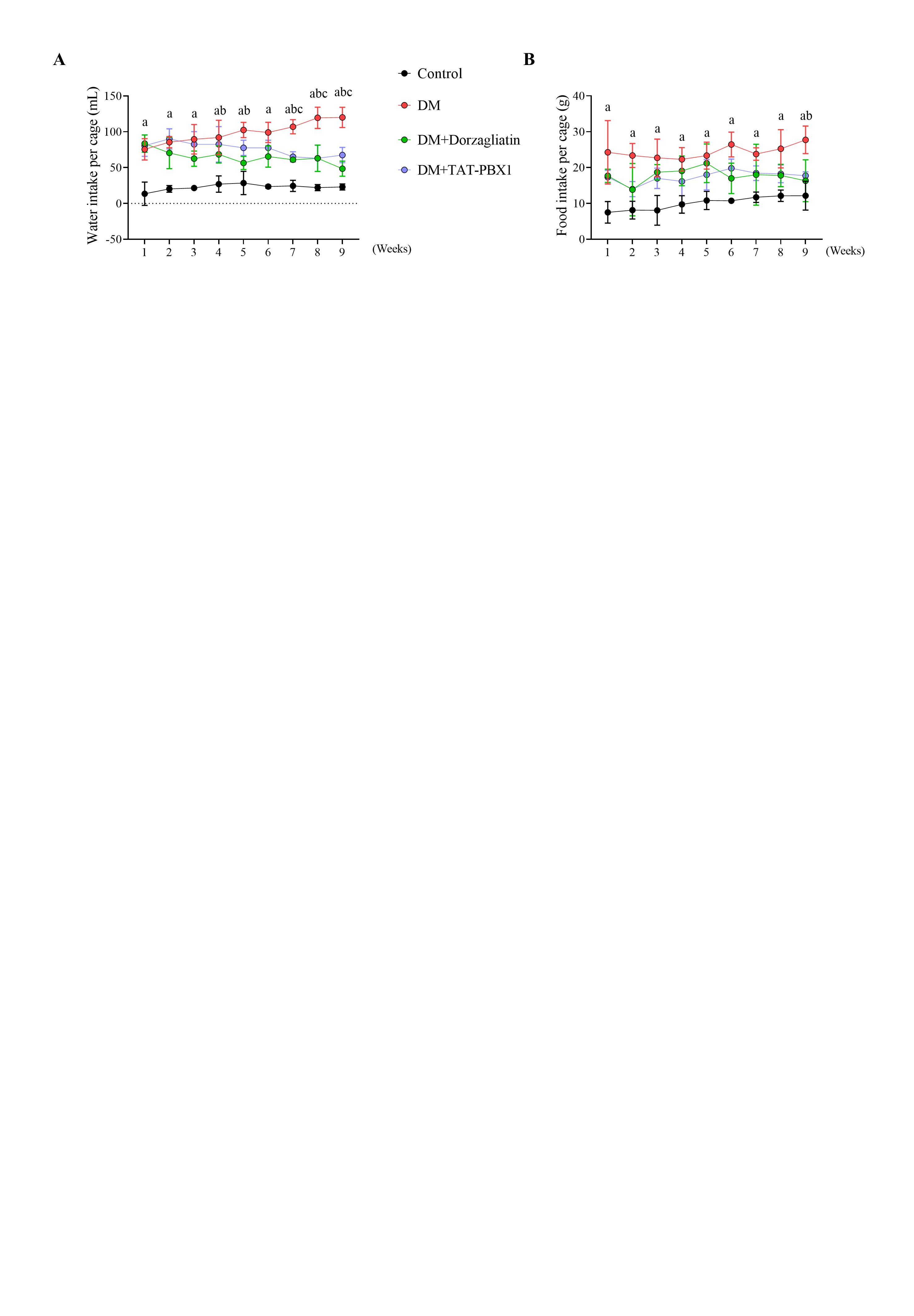

Supplement: Supplementary file 1 [file pharmaceuticals-19-00085-s001.zip › Figure S3.TIF]
